# Clinical Effectiveness of Total Hip Arthroplasty Compared With Hemiarthroplasty in Adults Undergoing Surgery for Displaced Intracapsular Hip Fracture: A Single-Centre Retrospective Cohort Study

**DOI:** 10.7759/cureus.45807

**Published:** 2023-09-23

**Authors:** Dhanushan Gnanendran, Yanamrigesh Yanaganasar, Jayagautham M Rajan, Zulkifli B Hassan, Neshvinder Balbir Singh, Lau Min Yi, Mohamed F Nadzree

**Affiliations:** 1 Trauma and Orthopaedics, York General Hospital, York, USA; 2 Geriatrics, Lincoln County Hospital, Lincoln, GBR; 3 Trauma and Orthopaedics, Bronglais General Hospital, Aberystwyth, GBR; 4 Trauma and Orthopaedics, Hospital Sultan Ismail, Johor Bahru, MYS; 5 Trauma and Orthopaedics, Newcastle University Medicine Malaysia, Johor Bahru, MYS

**Keywords:** geriatrics and internal medicine, orthopaedic arthroplasty, total hip arthroplasty, hemiarthroplasty, hip fracture

## Abstract

Background: The National Institute for Health and Care Excellence (NICE) recommends offering total hip arthroplasty (THA) over hemiarthroplasty (HA) for displaced intracapsular hip fractures, taking the premorbid functionality, present co-morbidities, and functional benefit beyond two years into account. Concerns remain whether the higher surgical burden and incidence of complications in THA would outweigh the potential benefits in the elderly.

Method: This retrospective cohort study evaluates the differences in surgical outcomes of THA vs HA in 85 patients with displaced intracapsular fractures, based on the time taken for patients to ambulate to walking frame/crutches and wheelchair post-operatively and the incidence of post-operative complications.

Results: Patients who received HA were significantly older (p<0.0001, <0.05) and had poorer pre-operative ambulatory function (p=0.032, p<0.05) than those of the THA group. HA patients had a significantly faster recovery to walking frame/crutches (20.2 days) compared to THA patients (47.3 days) (Mann-Whitney U=447.500, n=46, p=0.043, <0.05 two-tailed). While no significant differences were found in deep vein thrombosis (DVT), infected prosthesis, or dislocation incidence, hospital-acquired pneumonia (HAP) was more prevalent in THA patients (p=0.044, <0.05). Time to the walking frame had a significant effect on DVT/PE (p<0.001, <p0.05), the infected prosthesis (p=0.002, <0.05), and HAP (p=0.066, <0.05) incidence, while time to a wheelchair only affected HAP incidence (p<0.001, <0.05).

Conclusion: HA patients showed favourable outcomes in time to ambulate post-operatively and incidence of HAP among patients with advanced age and those with poorer pre-operative ambulatory function.

## Introduction

Hip fractures are a common occurrence globally, with an estimated incidence of 182 per 100,000 worldwide, in predominantly elderly and female patients [1]. Hip fractures are classified as intracapsular (femoral head/femoral neck) or extracapsular (intertrochanteric, trochanteric, or subtrochanteric). Hip fractures in the elderly are typically due to low-impact injuries and are often associated with osteoporosis. In contrast, high-impact injuries, such as motor vehicular accidents, prevail superior as the aetiology in younger patients. The intracapsular neck of femur fractures can be further classified by the Garden classification. The Garden classification considers displacement, fracture completeness, and relative misalignment of bony trabeculae between the femoral head and neck categorizing a hip fracture into one of four types [2]. The surgical management of choice for displaced intracapsular hip fractures remains a contentious subject.

Currently, surgical treatment generally involves arthroplasty broadly divided into total hip arthroplasty (THA) or hemiarthroplasty (HA). Both procedures incur risks to patients, including bleeding, infections, damage to vessels or nerves, venous thromboembolisms, and dislocated implants, which may result in poor functional recovery, prolonged hospitalisation, need for further operations, anaesthetic risks, and death. Surgical risks are broadly classified into risks due to the femoral components (e.g. loosening of prosthesis, dislocation, leg length discrepancy, etc.) and those unrelated to the femoral component (e.g. thrombosis, embolism, neurological disturbances). Recent studies have demonstrated the superior clinical outcomes of THA compared to HA [3].

However, the HA procedure is generally less extensive than the THA procedure. In addition to functional outcomes, surgeons would have to consider surgical extensiveness and incidence of complications when deciding on the surgical method for treating femoral neck fractures.

The ideal surgical method of choice in managing displaced intracapsular hip fractures in different population groups continues to be disputed, owing to the inconsistent findings of various studies. The choice of surgery would largely depend on surgeon preference and patient factors, including age, co-morbidities, and pre-operative mobility functional status [4].

The National Institute for Health and Care Excellence (NICE) currently recommends patients without any contraindication comorbidities with a previously independent ambulatory status and are expected to remain independently mobile for two or more years to be suitable candidates for THA instead of HA [5]. Studies have indicated that older, frail patients with poor pre-operative mobility status are preferential candidates for HAs, while younger and healthier counterparts are to be treated with THA [6,7]. On the contrary, a meta-analysis conducted on outcomes for THA vs HA concluded that THAs tend to have better functional improvement with reduced need for surgical revision among elderly patients [8].

Other studies have found insignificant evidence of differences in clinical improvement and post-operative complication rates between both procedures [9]. Studies have also found that post-operative complications are influenced primarily by patient factors, instead of the choice of procedure itself [10]. Ultimately, recommendations emphasise the importance of early surgery and coordinating care through a multidisciplinary team to help patients recover faster and regain their mobility.

The study aims to evaluate the differences in surgical outcomes of THA and hip HA by comparing the time taken to return to pre-operative ambulation and the incidence of post-operative complications in hip fracture patients undergoing THA vs HA over a span of 12 months in a Malaysian tertiary hospital.

Secondly, this study aim to evaluate any significant difference in the incidence of post-operative complications in patients undergoing THA vs HA over 12 months post-operation.

## Materials and methods

Inpatient and outpatient progress notes found in digitalised medical records in a Malaysian tertiary healthcare centre were used to gather data retrospectively. A total of 85 patients admitted for hip fractures having undergone THA and HA from 2017 to 2021 with a minimum follow-up record of 12 months were selected. Data recorded, including patient demographics, clinical comorbidities, length of inpatient stay, functional status, post-operative functionality, and incidence of post-operative complications, were obtained accordingly and tabulated. The presence of type 2 diabetes mellitus and hypertension and evidence of osteopenic/osteoporotic bone picture were defined under clinical comorbidities. As per hospital documentation protocols, pre-operative ambulatory functional status was classified as either ‘independent’ (being able to ambulate without assistance or with the use of a walking frame or crutches) or ‘dependent’ (being able to ambulate with a wheelchair). Garden classification for patients was extracted from the initial radiograph taken at presentation. Exclusion criteria were defined as those patients without 12 months of post-operative progress notes, patients with end-stage renal disease, patients with joint arthroplasty (upper and lower limbs), patients with arthropathies (osteoarthropathy and rheumatoid arthritis), and patients that incurred pre-operative/intra-operative mortality.

Return to pre-operative functionality was identified and compared among both groups of patients. The time to walking frame/crutches and the time to a wheelchair were used as measures to ensure the return to pre-operative mobility in the ‘ADL-independent’ and ‘ADL-dependent’ groups, respectively. To further assess the post-operative outcomes of the two procedures, the incidence of post-operative complications, namely, dislocated hip prosthesis, infected prosthetics, hospital-acquired pneumonia (HAP), and venous thromboembolic events (deep vein thrombosis and pulmonary embolism), were recorded.

Statistical analysis of the gathered data was performed using the Statistical Package for the Social Sciences (SPSS) software (IBM SPSS Statistics for Windows, Armonk, NY). Data were entered into Excel and analysed using SPSS 27 for Windows. Metric variables were expressed as mean/standard deviation, while categorical variables were expressed as count/percentage. Univariate analyses were performed using Pearson’s chi-square test for categorical variables. Kolmogorov-Smirnov and Shapiro-Wilkov tests were used to assess the normality of metric variables. Non-normal variables were then compared using the Mann-Whitney U test. Unadjusted p-values were then calculated, and a p-value of less than 0.05 (p<0.05) was considered statistically significant. The study was approved by the institutional ethical committee.

## Results

The demographic data show that the majority of patients who underwent THA originated from a younger age group with a mean of 68 years compared to the HA group's mean of 77 years (t-test: p<0.00001, <0.05). The majority of the patients were female n=66 (77.6%) and a higher proportion of male patients in the HA group (n=10, 11.8%). The demographic characteristics of our study cohort are presented comprehensively in Table 1. 

**Table 1:** Patient demographics. Independent ambulators = able to ambulate fully without support or aid. Dependent ambulators = requiring physical support or walking aids to ambulate. HA = Hemiarthroplasty, THA = Total Hip Arthroplasty Data have been represented as n (%).

		HA	THA
Total patients (n)		53 (62.4%)	32 (37.6%)
Mean age (years)		77	68
Gender	Males (n)	10 (11.8%)	9 (10.6%)
	Females (n)	43 (50.6%)	23 (27.1%)
Co-morbidities	Osteoporosis (n)	9 (10.6%)	5 (5.9%)
Pre-operative ambulatory function	Independent (n)	48 (56.4%)	31 (36.5%)
	Dependent (n)	5 (5.9%)	1 (1.2%)

Patients with osteoarthropathy or rheumatoid arthritis were excluded from the study as the return to function of such patients would have been significantly affected by the presence of large/small joint arthropathies. Despite a large proportion of patients being ‘independent’ in ambulatory function (n=79, 92.9%), the HA group consisted of more ‘dependent’ patients (n=5, 5.9%) compared to the THA group (n=1, 1.2%), with a significant difference found via a t-test (p=0.0322, <0.05).

Of the total patients who underwent THA, only one patient was found to be ambulating in a wheelchair pre-operatively (Table 2). This patient had suffered an alleged fall several weeks prior to the THA being performed, resulting in him being wheelchair-bound pre-operatively. With that patient excluded, the remaining patients were found to have a mean time to walking frame/crutches of 47.3 days. In the THA group, two patients failed to ambulate with a walking frame/crutches post-operatively within 12 months.

**Table 2:** Mean times taken to recover from the ambulatory function post-operatively to a wheelchair and walking frame/crutches. Note: Patients who did not return to a wheelchair and walking frame/crutches within one year of follow-up were excluded from the calculated mean times. Ambulation times, which were not accurately documented, were also excluded. HA = Hemiarthroplasty, THA = Total Hip Arthroplasty Data have been represented as a mean.

	HA	THA
Mean time to a wheelchair (days)	4.8	4.5
Mean time to a walking frame/crutches (days)	20.2	47.3
Number of patients failed to ambulate to a walking frame/crutches	1 (1.9%)	2 (6.3%)

In the HA group, five patients were ambulating in a wheelchair pre-operatively. These patients had a multitude of reasons for their limited pre-operative mobility. These included L4/L5 spondylodiscitis, congenital deformities of the foot, and post-wound debridement, as well as two patients with co-existing fractures (patellar and distal tibial) at the time of presentation. With those patients excluded, the remaining patients were found to have a mean time to a walking frame/crutches of 20.2 days. In the HA group, only one patient failed to ambulate to a walking frame/crutches post-operatively within 12 months.

Normality tests were performed on the time taken to return to post-operative functional status, which revealed the data as non-normally distributed. Mann-Whitney U tests were performed to evaluate the difference in the post-operative return to function between the two procedures.

THA (27.85) had a lower mean rank than HA (33.24) for the time taken to return to the wheelchair (‘Time to a wheelchair’) post-operatively. There is no significant difference in ‘Time to a wheelchair’ post-operation between THA and HA (Mann-Whitney U=493.000, n=39, p=0.265, >0.05 two-tailed). With regard to ‘Time to walking frame/crutches’ post-operation, THA (42.57) had a higher mean rank than HA (32.41). Post-operatively, a significant difference was found in ‘Time to walking frame/crutches’ between THA and HA (Mann-Whitney U=447.500, n=46, p=0.043, <0.05 two-tailed).

With regard to complications, dislocations (n=5, 16%), HAP (n=4, 12.5%), and infected prosthetics (n=4, 12.5%) were more common amongst the THA group, while the incidence of DVT/PE (n=2, 3.8%) was higher in the HA group. Chi-square analysis was performed to evaluate the differences in DVT, HAP, dislocations, and infected prosthesis between THA and HA groups, as shown in Table 3.

**Table 3:** Results chi-square analysis comparing the incidence of complications between total hip arthroplasty and hemiarthroplasty. HA = Hemiarthroplasty, THA = Total Hip Arthroplasty, DVT = Deep Vein Thrombosis, PE = Pulmonary Embolism, HAP = Hospital Acquired Pneumonia Data have been represented as n. p-value of less than 0.05 was considered statistically significant.

Complications		Procedure		Chi-square value	P-value
		THA	HA		
Dislocations	Absent	27	50	2.324	0.127
	Present	5	3		
DVT/PE	Absent	32	51	1.237	0.266
	Present	0	2		
Infected prosthesis	Absent	28	50	0.420	0.517
	Present	4	3		
HAP	Absent	28	52	4.060	0.044
	Present	4	1		
Complication-related mortality	Absent	29	52	2.495	0.114
	Present	3	1		

There is no significant difference in the incidence of dislocations between the two procedures (p=0.127>0.05), and there is no significant difference in the count of DVT/PE between the two procedures (p=0.266>0.05). Moreover, there is no significant difference in the count of infected prostheses between the two procedures (p=0.517>0.05). However, HAP is significantly different between the two procedures (p=0.044<0.05).

Despite the higher mortality in THA than HA (9.4% (n=3) of THA patients versus 1.9% (n=1) of HA patients), the chi-square analysis proved that the difference was not significant (p=0.114>0.05). Further analysis performed to assess the impact of time taken to ambulate with a walking frame/crutches and a wheelchair on the incidence of complications is recorded in Table 4.

**Table 4:** Results of chi-square analysis to assess the impact of the time to a walking frame/crutches and time to a wheelchair on the incidence of post-operative complications. HA = Hemiarthroplasty, THA = Total Hip Arthroplasty, DVT = Deep Vein Thrombosis, PE = Pulmonary Embolism, HAP = Hospital Acquired Pneumonia p-value of less than 0.05 was considered statistically significant.

Complication	Time to walking frame/crutches		Time to wheelchair	
	Chi-square value	P-value	Chi-square value	P-value
Dislocations	34.10	0.321	15.39	0.283
DVT/PE	72.00	<0.001	4.70	0.981
Infected prosthesis	59.48	0.002	2.52	0.999
HAP	43.63	0.066	36.04	0.001

‘Time to a walking frame/crutches’ had a significant impact on DVT/PE (p<0.001, <0.05), the infected prosthesis (p=0.002, <0.05), and HAP (p=0.066, <0.05). On the other hand, ‘Time to a wheelchair’ only significantly affected the incidence of HAP (p<0.001, <0.05). It was not surprising to observe that a faster ambulation rate correlated with a reduced incidence of complications.

## Discussion

When comparing the patients treated with HA and THA, there was a statistically significant difference in the age and pre-operative ambulatory function, whereby patients treated preferentially with HA were older and of poorer pre-operative ambulatory function, which largely aligns with the recommendations of offering a total hip replacement, rather than hemiarthroplasty, for people with displaced intracapsular hip fractures as outlined by NICE [5].

In comparing the functional outcome between THA and HA, the results showed HA patients were able to ambulate with a walking aid significantly faster than the THA patients, while wheelchair ambulation time was comparable between both procedures. It is important to consider that post-operative protocols significantly influence the duration of wheelchair ambulation post-operatively, which could not be accounted for in this study. While there was a higher proportion of patients who underwent HA failing to ambulate with a walking frame post-operatively, four out of the seven patients who were pre-operatively ‘dependent’ ambulators were able to recover their function to a walking frame/crutches post-operatively.

Multiple studies have found THA to be superior in pain and functional outcomes after several years post-operatively and generally recommend it in patients with an active pre-morbid status [7,11,12]. A more recent outcome analysis also showed THA to possess better functional outcomes at one-year follow-up, despite a higher post-operative complication rate [13]. Furthermore, the rapid recovery of function seen in HA patients is likely attributed to the reduced amount of trauma incurred by the procedure, which is less extensive than in THA. Despite the evidence of superiority in functional outcomes of THA, studies are still unable to draw conclusive recommendations as the benefits in different age groups are seldom assessed adequately due to selection bias, which generally excludes less active and elderly patients from receiving THA. Randomised controlled trials (RCTs) have acknowledged this limitation, which prevents true randomisation of procedures offered to patients [12].

Both procedures were identical in complication rates, except for the higher incidence of HAP in THA. This could be partly attributed to the prolonged time taken to ambulate in the THA group, as the data showed that both ambulation with a wheelchair and walking frame had a significant influence on the incidence of HAP.

This largely aligns with the literature as multiple studies found the rates of complications to be identical between both procedures [11,13,14]. It has been noted that THAs have led to higher dislocation rates and a predictable higher reported blood loss, but also result in lower reoperation rates [8,15]. On the contrary, one RCT showed THA to have lower complication rates than HA [16]. It is also important to note that the duration spent in the operation theatre is another factor that can affect the incidence of complications amongst the THA and HA groups. THA had an average duration of 2.4 hours, while HAs were completed within an average of 2.2 hours. As such, the discrepancy was not sufficient to account for the difference in the rate of complications in this study.

Given that this study was conducted retrospectively, this introduces several limitations on the reliability of the outcomes produced. The patients involved were not assigned to the assessed hip arthroplasty at random and were instead managed based on the clinical judgment of the surgical team involved in their care and the local guidelines practised at the hospital. The retrospectively collected patient notes had not been purposefully documented with a standardised data collection system, and the measurements of functional outcomes were restricted as no standardised scoring tools, which may have been used to assess pre-operative and post-operative function, had been documented. Despite the hospital using a qualitative approach in categorising functional status both pre-operatively and on follow-up visits, the use of a standardised quantitative tool to measure patient functionality would ensure that the return to baseline functional mobility is more concrete.

The limited sample size further restraints the reliability of the results since NICE has recommended that a randomised RCT with a sample size of at least 500 patients is needed to achieve conclusive results [5]. Further, RCTs with a larger sample size are required to confirm these findings and to investigate the influence of revision surgery and dislocations on mortality [10]. Other areas not addressed by this study include the cost-effectiveness and improvement in quality of life to allow for the efficient utilisation of funds in a large population. Outcomes between various prosthesis types and manufacturers should also be accounted for.

## Conclusions

While no conclusive recommendations can be derived, HA, as a treatment for displaced intracapsular hip fractures, has shown favourable outcomes with regard to post-operative recovery of ambulatory function and complication rates among older patients with significantly poorer pre-operative ambulatory function. High-quality RCTs are required to derive accurate guidelines for the surgical management of displaced intracapsular hip fractures.
